# Fringe-fields-modulated double-gate tunnel-FET biosensor

**DOI:** 10.1038/s41598-023-50723-y

**Published:** 2024-01-02

**Authors:** Iman Chahardah Cherik, Saeed Mohammadi

**Affiliations:** Department of Electrical and Computer Engineering, Semnan University, Semnan, 3513119111 Iran

**Keywords:** Nanobiotechnology, Nanoscale devices, Electrical and electronic engineering

## Abstract

This paper aims to evaluate a groundbreaking bio-TFET that utilizes the fringe fields capacitance concept to detect neutral and charged biomolecules. While facilitating fabrication process and scalability, this innovative bio-TFET is able to rival the conventional bio-TFET which relies on carving cavities in the gate oxide. The cavities of the proposed device are carved in the spacers over the source region and in the vicinity of the gate metal. Inserting biomolecules in the cavities of our bio-TFET modifies the fringe fields arising out of the gate metal. As a result, these spacers modulate tunneling barrier width at the source-channel tunneling junction. We have assessed our proposed device’s DC/RF performance using the calibrated Silvaco ATLAS device simulator. For further evaluation of the reliability of our bio-TFET, non-idealities, such as trap-assisted tunneling and temperature, are also studied. The device we propose is highly suitable for biosensing applications, as evidenced by the parameters of ${S}_{{I}_{ds}}$ = 1.21 × 10^3^, *S*_*SS*_ = 0.365, and ${S}_{{f}_{T}}$ = 1.63 × 10^3^ at *V*_*GS*_ = 1 V.

## Introduction

The demand for high-performance biosensors which can detect various diseases in their early stages is rapidly increasing. Label detection-based biosensors fall short of sensitivity and can alter the intrinsic characteristics of biomolecules^1,2^. In contrast, dielectric-modulated label-free biosensors preserve natural characteristics of biomolecules^3^. The ion-sensitive field effect transistor (ISFET) was the first label-free biosensor based on FETs, but it could only detect charged biomolecules^4^. To address this limitation, researchers developed the dielectric-modulated field-effect transistor (DMFET) to detect charged and neutral biomolecules^5^. The DMFET contains cavities carved in the gate metal or gate oxide of a MOSFET, where the entrance of biomolecules changes the electrostatic control of the gate, leading to the change in different device’s electronic characteristics. However, shrinking the dimensions of MOSFETs into the nanometer regime causes crucial problems for biosensing applications of DMFETs^6^.

Tunneling field-effect transistors (TFETs) are being considered as a potential alternative to short-channel MOSFETs. Unlike MOSFETs, TFETs regulate subthreshold swing through electrostatic control of the gate. TFETs also benefit from band pass filtering of high-energy electrons in the source region, leading to a much lower off-state current than that of MOSFETs^7^. Furthermore, the TFETs’ major limitations, such as low on-state current and ambipolar conduction, have been effectively addressed (see^8–14^).

Several TFET-based biosensors with various geometry have been proposed. Some of the most interesting introduced devices are double-gate^15,16^, vertical^17–20^, core-shell nanotube^21–23^, charge-plasma^24–28^, and electron–hole bilayer^29^ TFET structures. However, in all of these devices, the gate leakage current can affect the performance of the biosensor. To address this issue, some researchers have suggested using a low-thickness sacrificial layer between the gate and channel regions^30^, but this approach can create challenges during the fabrication process.

This paper suggests a novel double-gate biosensor in which two cavities are carved over the source region. With applying the gate voltage, fringe fields in the spacers modify the strength of the electric field at the source-channel junction, resulting in considerable variations in the energy barrier width of the tunneling window. By employing a low-defect Si-SiO_2_ interface as the semiconductor-oxide junction, our proposed bioTFET not only benefits from full compatibility with CMOS technology but also ensures high reliability. Moreover, the fabrication process is less arduous compared to bio-TFETs that rely on nanowires or nanotubes configurations. Additionally, the non-etched gate oxide and asymmetric doping eliminates leakage current and ambipolar conduction, respectively.

## Device structure, fabrication process, and simulation methodology

**Figure 1:**
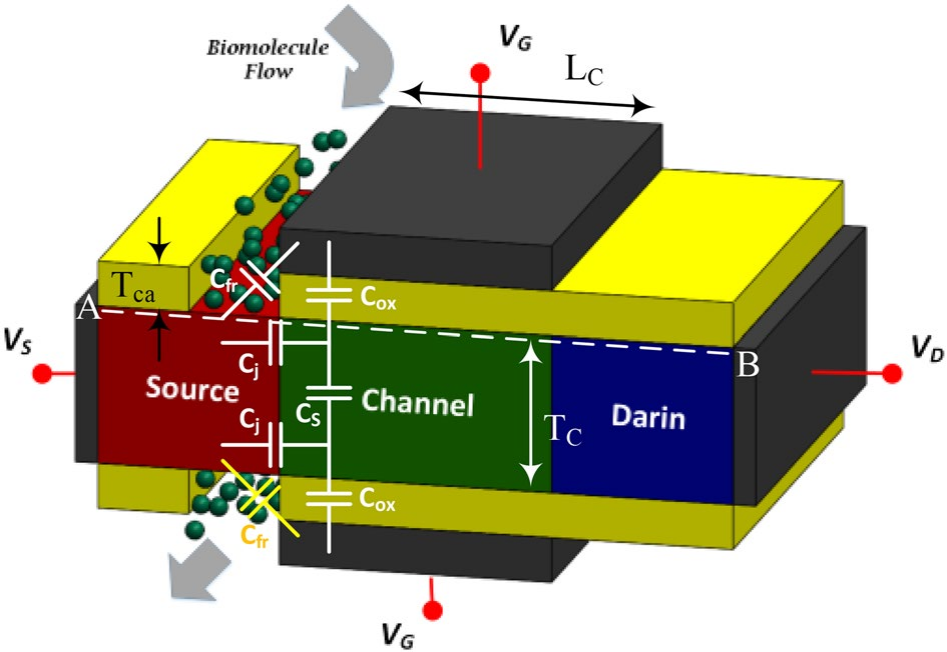
A schematic view of the proposed FFC-bioTFET structure along with a simple capacitive model.

A schematic two dimensional view of our designed bio-sensor (named FFC-bioTFET) is shown in Fig. 1. To have the most compatibility with CMOS technology conventional Si-SiO_2_ structure is employed. In this device the source, channel, and drain regions are doped with the impurity concentrations of 1 × 10^19^ cm^−3^, 1 × 10^17^ cm^−3^, and 3 × 10^18^ cm^−3^, respectively. In order to convert the double-gate TFET into a biosensor, two cavities measuring 20 nm × 7 nm are created in the source side spacers. Compared to the conventional DMFET biosensors which need a sacrificial layer to prevent gate-leakage current, in our bioTFET no such a layer is necessary. Reducing fabrication challenges is another benefit of carving cavities in the spacer regions. To prevent direct source-to-drain tunneling, we used a 50 nm channel between the source and drain. Metal gates, with a work function of 4 eV, modulate the energy bands at the tunneling junction. This figure also includes a simple capacitive model of the proposed biosensor at the tunneling junction. It can be inferred from the model that by inserting biomolecules with higher permittivity, which can be modeled by increased *C*_*fr*_, the electric field across *C*_*j*_, which models the tunneling junction, reduces and consequently the tunneling rate degrades.

**Figure 2:**
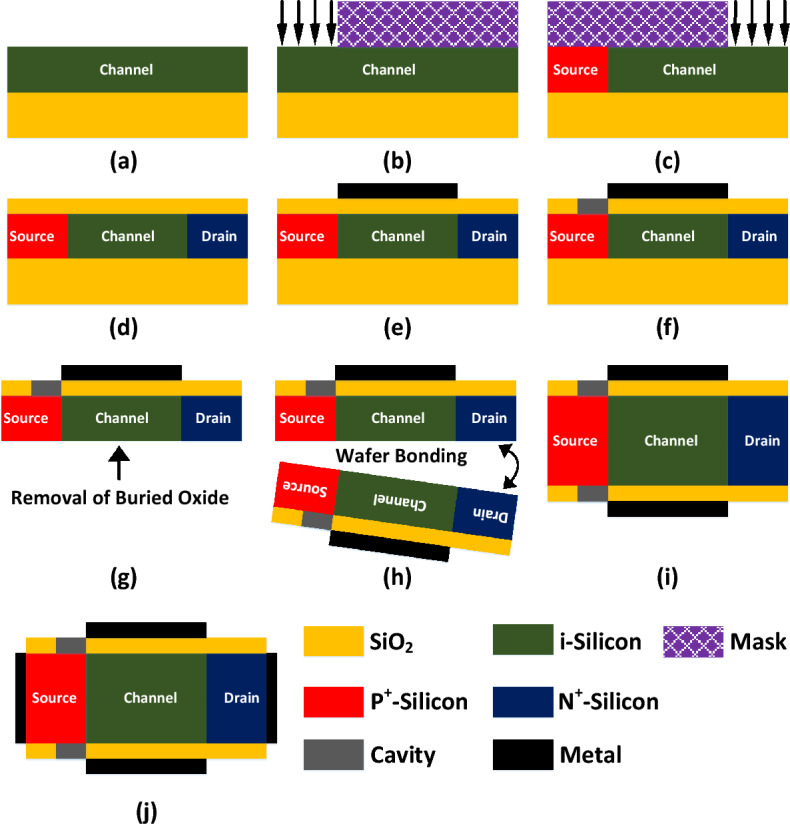
(**a–j**) Fabrication process steps for realizing FFC-bioTFET structure.

Figure 2 presents a clear and precise fabrication process for FFC-bio TFET. To fabricate the biosensor, two wafers with the same fabrication procedures should be prepared. In the first step, an SOI wafer is created (see Fig. 2a). Then, p^+^-source and n^+^-drain are created using two consecutive ion-implantation steps (see Fig. 2b,c). After the deposition of the SiO_2_ as the spacer, gate metal is deposited (see Fig. 2d,e). In the next step, the cavity is carved in the spacer using the wet-etching technique, followed by removing the buried oxide (See Fig. 2f,g). Then, two wafers are bonded together (see Fig. 2h,i). In the last stage, source and drain contacts are connected (see Fig. 2j).

**Figure 3:**
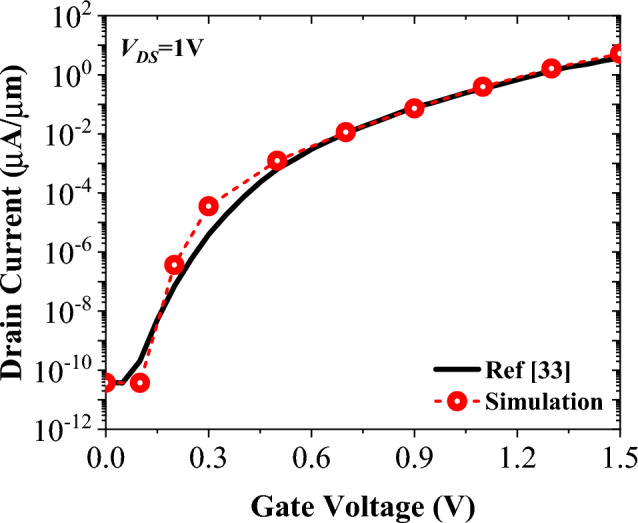
Reproduction of the transfer characteristic of Ref.^33^ by our calibrated simulation framework.

Our proposed biosensor is simulated by using the Silvaco ATLAS device simulator^31^, where the following models are activated to generate different device characteristics. The dynamic nonlocal band-to-band tunneling model is enabled to calculate the current at the source-channel tunneling junction. Defects at the semiconductor-oxide interface can deteriorate the performance of TFET-based biosensors, so we have used the Interface model with the *D*_*it*_ = 3 × 10^11^ cm^−2^ eV^−1^ for all simulations^32^. Since silicon has a large indirect bandgap and channel thickness is 10 nm, subband quantization is not considered. The gate leakage current was not taken into account because 7 nm SiO_2_ was used as the gate oxide. All other activated models are SRH, auger, BGN, fermi, and CVT. The transfer characteristics of the simulated device is plotted and compared to the extracted values of the Ref.^33^. The depicted results of Fig. 3 demonstrate a good match, effectively validating the simulation procedure.

## Simulation results

**Figure 4:**
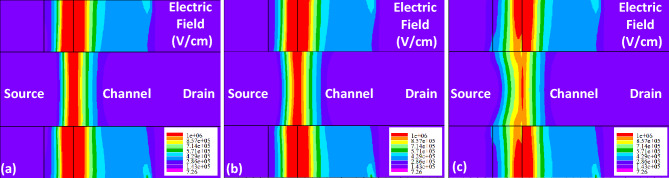
Electric field contour maps at the tunneling junction of FFC-TFET for (**a**) k = 1, (**b**) k = 3.57, and (**c**) k = 12.

In this section, we will analyze how different neutral and charged biomolecules affect the AC/DC performance of FFC-bio TFET. Additionally, we will assess the reliability of our proposed structure under non-ideal conditions, such as temperature variation and trap states. Figure 4 depicts the electric field contours of the device when the cavities are filled by Air, APTES (k = 3.57) and Gelatin (k = 12), respectively. It can be inferred that insertion of biomolecules with higher dielectric constants in the cavities increases the extension of the electric field lines in the source region and consequently smooths the lateral electric field at the tunneling junction which leads to a longer tunneling path for the charge carriers.

**Figure 5:**
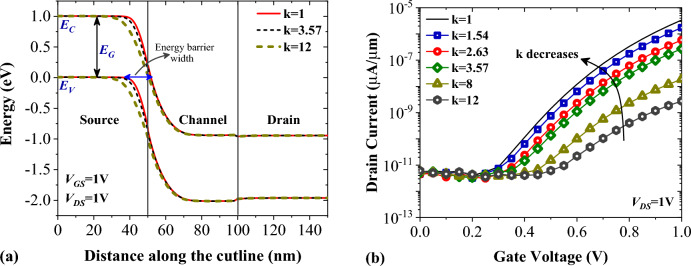
Impact of different neutral biomolecules on (**a**) the energy band diagram, and (**b**) the transfer characteristics of FFC-bioTFET.

Figure 5a illustrates the impact of the dielectric constant of air and biomolecules on the energy bands diagram of FFC-bioTFET along the $\overline{{\text{AB}} }$ segment (as shown in Fig. 1). We can see that while the modulation of the energy bands in the channel is negligible, biomolecules with higher dielectric constant significantly modulate the band bending in the source region, as a result tunneling barrier width increases. It can be seen that at the *V*_*GS*_ = 0.5 V, the value of the energy barrier width for Air and Gelatin is 25.6 nm and 36.3 nm, respectively. With the doubling of gate voltage, the value of the abovementioned parameters decreases to 17.8 nm and 27.1 nm, respectively. In Fig. 5b, the impact of various neutral biomolecules on the transfer characteristics of FFC-bioTFET is depicted. In conventional bio-TFET, biomolecules with higher dielectric constants enhance the band-to-band tunneling rate at the source-channel junction. In FFC-bioTFET, we have an inverse scenario, meaning that with increasing biomolecule’s dielectric constants drain current decreases.

**Figure 6:**
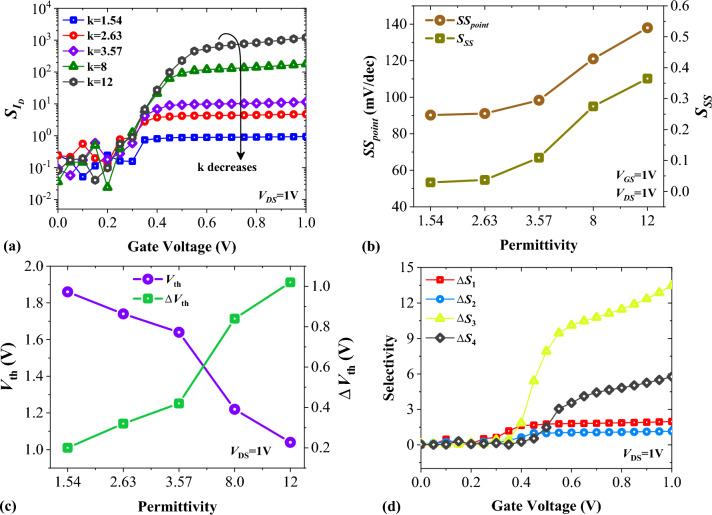
Impact of different neutral biomolecules on (**a**) the drain current sensitivity, (**b**) the subthreshold swing and subthreshold swing sensitivity of FFC-bioTFET, and (**c**) the threshold voltage, and threshold voltage sensitivity of FFC-bioTFET. (**d**) Selectivity of FFC-bioTFET between different pairs of neutral biomolecules.

In Fig. 6a, the impact of the biomolecule’s dielectric constant on the drain current sensitivity of our proposed structure is depicted. It is given by ${S}_{{I}_{D}}=\left(\frac{{I}_{ds}^{air}-{I}_{ds}^{bio}}{{I}_{ds}^{bio}}\right)$; here, ${I}_{ds}^{air}$ is the drain current when we use Air in the cavities and is the constant part of the relationship, while the values of ${I}_{ds}^{bio}$ highly depend on the type of biomolecules we use in the cavities. For k = 1.54, we have a ${S}_{{I}_{ds.max}}$ = 0.94, while having Gelatin in the cavities causes this to reach to 1.21 × 10^3^ (which indicates more than three decades increase). The performance of a bio-TFET can be assessed by considering its subthreshold swing (*SS*) and subthreshold swing sensitivity (*S*_*SS*_), too. Figure 6b shows the impact of various neutral biomolecules on the *SS* and *S*_*SS*_ of FFC-bioTFET. Subthreshold swing sensitivity is defined by ${S}_{SS}=\left|\frac{{SS}_{bio}-{SS}_{air}}{{SS}_{bio}}\right|$, where *SS*_air_ and *SS*_bio_ are the minimum gate voltages required for a ten-time increment of the drain current in the presence of Air and biomolecule, respectively. By using Gelatin instead of Uricase (k = 1.54), the value of *SS* and *S*_*SS*_ reaches from 90.23 mV/dec and 0.029 to 137.99 mV/dec and 0.365, respectively. Figure 6c illustrates how the presence of neutral biomolecules affects the threshold voltage and the threshold voltage sensitivity of FFC-bioTFET. To obtain the threshold voltage we employed the maximum transconductance method as a more reliable threshold voltage measure^34^. We have defined the threshold voltage sensitivity as Δ*V*_*th* =_ *V*_*th*(*air*)_ − *V*_*th*(*bio*)_, where *V*_*th*(*air*)_ is the threshold voltage for k = 1, and *V*_*th*(*bio*)_ is the threshold voltage in the presence of biomolecules in the cavities^21^.

In addition to excellent sensitivity, a good sensor should exhibit good selectivity. Selectivity is one of the fundamental parameters for a biosensor, and enables biosensor to identify a specific biomolecule in the present of another biomolecule. To have an evaluation about this parameter, we have calculated the selectivity between four pairs of natural biomolecules with the following relationships^35^,

$${\Delta S}_{1}=\frac{{I}_{ds,Uricase}-{I}_{ds,Biotin}}{{I}_{ds,Biotin}},$$

$${\Delta S}_{2}=\frac{{I}_{ds,Biotin}-{I}_{ds,APTES}}{{I}_{ds,APTES}},$$

$${\Delta S}_{3}=\frac{{I}_{ds,APTES}-{I}_{ds,Keratin}}{{I}_{ds,Keratin}},$$

$${\Delta S}_{4}=\frac{{I}_{ds,Keratin}-{I}_{ds,Gelatin}}{{I}_{ds,Gelatin}},$$

and the results are depicted in Fig. 6d. It can be observed that FFC-bioTFET reveals more efficient selectivity to detect between APTES and Keratin. This can be attributed to the higher values of *Δε*_*k*_/*ε*_*k*_ for the aforementioned pair of biomolecules compared to other cases.

**Figure 7:**
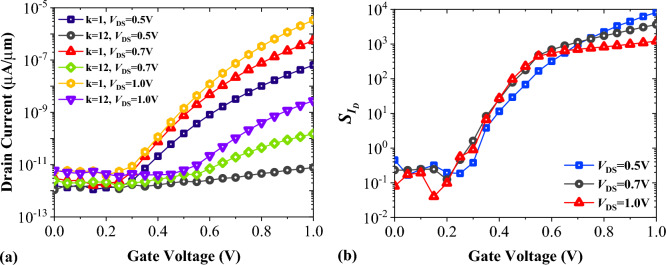
Impact of drain voltage on (**a**) transfer characteristics, and (**b**) drain current sensitivity of FFC-bioTFET.

In tunneling FETs, when *V*_DS_ is lower than *V*_GS_, raising the drain voltage significantly modulates the tunneling junction, increasing available states for tunneling. Figure 7a shows the impact of *V*_DS_ variation on the transfer characteristics of FFC-bioTFET for Air and Gelatin. It is evident that raising the *V*_DS_ leads to a higher on-state current for both cases. Figure 7b shows that with the reduction of *V*_DS_, drain current sensitivity increases.

Biosensors based on tunnel transistors can also detect a type of biomolecule that possess charge density known as charged biomolecules, hence, we investigate the impact of a well-known charged biomolecule called deoxyribonucleic acid (DNA) on the DC performance of our proposed biosensor.

**Figure 8:**
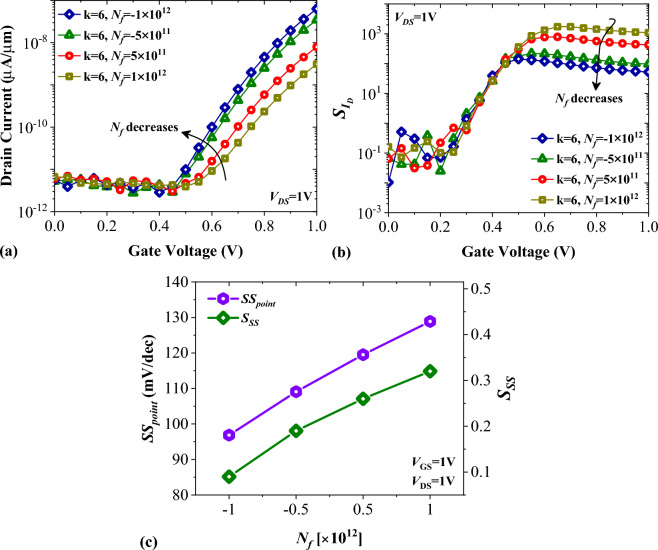
Impact of DNA biomolecules on (**a**) the transfer characteristics, (**b**) drain current sensitivity, and (**c**) subthreshold swing and subthreshold swing sensitivity of FFC-bioTFET.

In Fig. 8a, the transfer characteristics of FFC-bioTFET for four different charge densities (− 1 × 10^12^ C.cm^−2^, − 5 × 10^11^ C.cm^−2^, 5 × 10^11^ C.cm^−2^, and 1 × 10^12^ C.cm^−2^) reveal that the curves with positively charged biomolecules exhibit lower on-state currents when compared to negatively charged ones. Indeed, the positively charged biomolecules have adverse influence on the band bending sharpness at the source-channel tunneling junction. Figure 8b displays the impact of charged biomolecules on the drain current sensitivity of FFC-bioTFET. The drain–current sensitivity of positively charged biomolecules is higher than negatively charged ones since positively charged biomolecules degrade the on-state current more. For DNA biomolecule with *N*_*f*_  = − 1 × 10^12^ C.cm^−2^, ${S}_{{I}_{ds}}$ = 52.29, while for *N*_*f*_ = 1 ×  10^12^ C.cm^−2^, ${S}_{{I}_{ds}}$ with more than one decade increment reaches to 1.09 × 10^3^. In Fig. 8c, it is shown how the charged biomolecules of DNA affect the subthreshold swing (*SS*) and subthreshold swing sensitivity (*S*_*SS*_) of FFC-bioTFET. The lowest *SS* is observed for DNA biomolecules with *N*_*f*_ = − 1 × 10^12^ C.cm^−2^, while for *N*_*f*_ = 1 × 10^12^ C.cm^−2^, this value has a ~ 133% increase. However, the highest *S*_*SS*_ is observed for DNA with *N*_*f*_ = 1 × 10^12^ C.cm^−2^, as compared to the other three cases.

**Figure 9:**
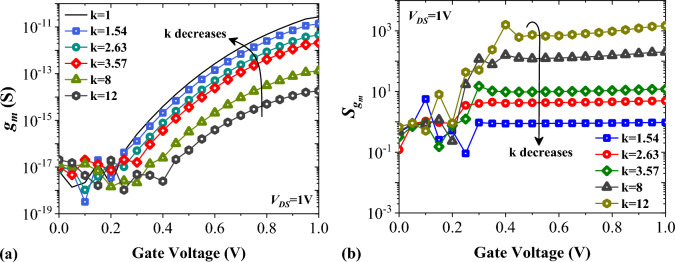
Impact of different biomolecules on (**a**) the transconductance, and (**b**) the transconductance sensitivity of FFC-bioTFET.

**Figure 10:**
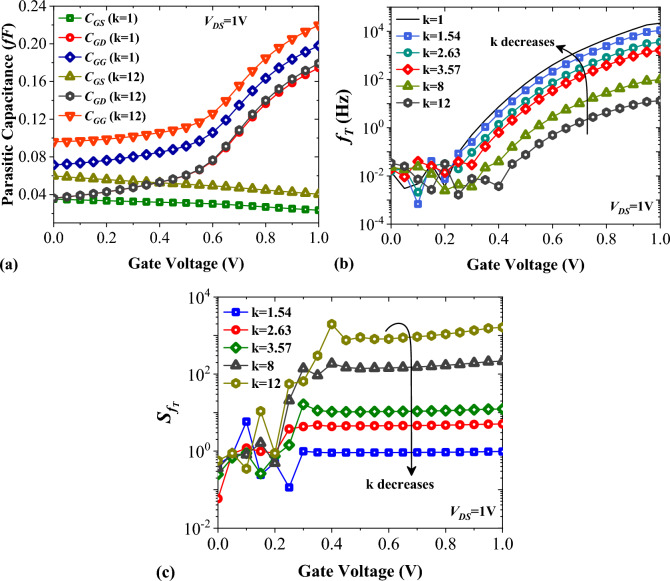
Impact of different biomolecules on (**a**) the parasitic capacitances, (**b**) the cut-off frequency, and (**c**) the cut-off frequency sensitivity of FFC-bioTFET.

Linearity is an important factor in highly sensitive biosensors. To evaluate the linearity of a biosensor, the transconductance must be calculated. The equation *g*_*m*_ = $\partial$*I*_*D*_/$\partial$*V*_*GS*_ determines this value^36^. Figure 9a shows that when the value of k changes from 1 to 12, the transconductance decreases due to the effect of higher dielectric constants on the band bending at the tunneling junction. We plotted the transconductance sensitivity as a function of the biomolecule’s dielectric constant in Fig. 9b. It is noticeable that biomolecules with higher values of k result in higher ${S}_{{g}_{m}}$. The parasitic gate-to-source and gate-to-drain capacitances significantly influence the AC performance of FETs. In this section, we have examined how these parameters impact the total gate capacitance (*C*_*GG*_), cut-off frequency (*f*_*T*_), and sensitivity of cut-off frequency (${S}_{{f}_{T}}$). Figure 10a shows the variation of the parasitic capacitances of FFC-bioTFET for Air and Gelatin molecules. When k = 12, the value of *C*_*GS*_ is slightly higher than when k = 1, since the higher k values increase the extension of fringe field lines. It is worth noting that gate-to-drain capacitance dominates the total gate capacitance for both cases, except when k = 12 and *V*_*GS*_ is lower than 0.4 V. In Fig. 10b,c, we see the cut-off frequency and its sensitivity changing as k varies between 1 and 12. The graph in Fig. 10b shows that higher values of k lead to lower *f*_*T*_, mainly because *g*_*m*_ decreases (as shown in Fig. 9a). Moreover, higher values of k contribute to the higher values of the total gate capacitance. According to Fig. 10c, ${S}_{{f}_{T}}$ varies as we change the biomolecule. We notice that using Gelatin instead of Uricase significantly increases ${S}_{{f}_{T}}$ by several decades, reaching from 0.97 to 1.63 × 10^3^.

**Figure 11:**
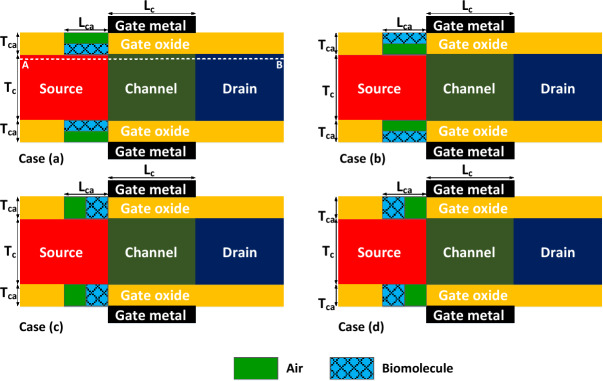
Various types of semi-filled cavities.

**Figure 12:**
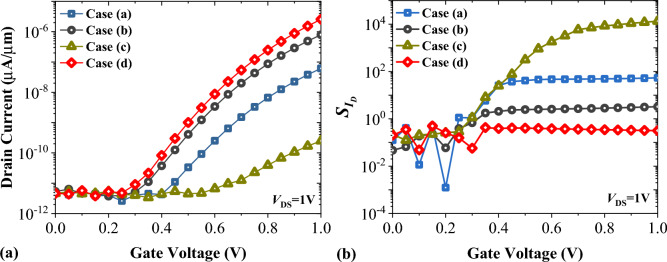
The impact of semi-filled cavities on (**a**) transfer characteristics, and (**b**) drain current sensitivity of FFC-bioTFET.

Non-uniform distribution of biomolecules in the biosensor’s cavities may affect the sensitivity of the device. In order to investigate the impact of non-ideal filling of cavities we have considered four different semi-filled profiles with a filling factor of 50% as depicted in Fig. 11. Gelatin biomolecule is considered as the sample in this investigation. Figure 12a demonstrates the impact of unfilled cavities on the transfer characteristics of FFC-bioTFET. It is noticeable that the on-state current in Case (d) surpasses the other cases. In fact, when the low-k spacer is closer to the source-channel junction, the energy bands of the source region are less affected by the fringe fields and consequently the on-state current is higher. Meanwhile, Fig. 12b illustrates that Case (c), in which the permittivity of the gate spacers is higher than that of the other three cases, exhibits the most heightened sensitivity of the drain current.

**Figure 13:**
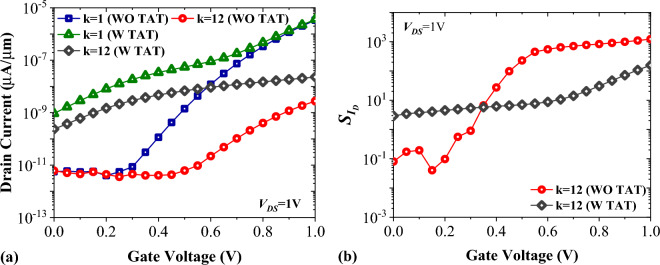
Impact of trap-assisted tunneling on (**a**) the transfer characteristics, and (**b**) the drain current sensitivity of FFC-bioTFET.

Trap-assisted tunneling is a detrimental phenomenon which shades a notable impact on the performance of TFETs^37^. Figure 13a shows that activating this model considerably increases the off-state current of FFC-bioTFET. Figure 13b portrays the impact of TAT on the ${S}_{{I}_{ds}}$ of FFC-bioTFET for k = 12. If the *V*_*GS*_ falls below 0.35 V, TAT will cause a reduction in drain current sensitivity. On the other hand, if TAT is not engaged and *V*_*GS*_ is above 0.35 V, we can expect higher ${S}_{{I}_{ds}}$ values.

**Figure 14:**
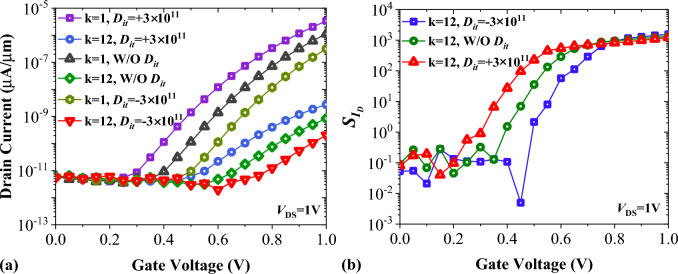
The impact of interface trap charges on (**a**) transfer characteristics, and (**b**) drain current sensitivity of FFC-bioTFET.

The other non-ideality that may significantly influence the TFETs performance is the impact of interface trap charges (ITCs). It should be emphasized that the oxide–semiconductor interface of our proposed structure is composed of SiO_2_-Si, which is the most desirable case in terms of interface trap density. In Fig. 14a we have shown that FFC-bioTFET with negative ITC poses the highest onset voltage for k = 1 and k = 12. As a result, the on-state current for *D*_*it*_ = − 3 × 10^11^ cm^−2^ eV^−1^ is lower than those of other cases. On the other hand, the onset of band-to-band tunneling of FFC-bioTFET for *D*_*it*_ =  + 3 × 10^11^ cm^−2^ eV^−1^ reduces for both cases. Figure 14b depicts the impact of interface trap charges on the drain current sensitivity of our structure. Similar to Fig. 14a, the positively charged interface of SiO_2_-Si displays better transduction efficiency for a wider range of gate voltages.

**Figure 15:**
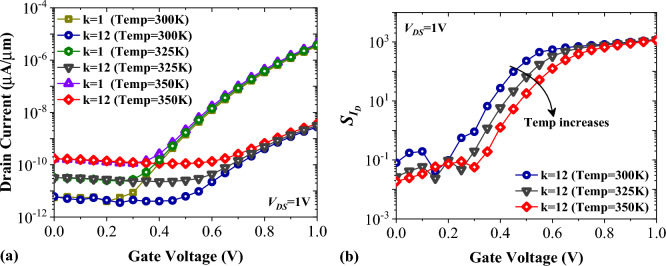
Impact of temperature on (**a**) transfer characteristics, and (**b**) drain current sensitivity of FFC-bioTFET.

The performance of TFETs in the off-state and subthreshold region is greatly impacted by changes in temperature. In Fig. 15a, we demonstrate the effects of increasing the temperature by 50 K on the transfer characteristics of FFC-bioTFET. Notably, the off-state current experiences a significant increase for both k = 1 and k = 12. Figure 15b depicts how temperature affects the ${S}_{{I}_{ds}}$ of our biosensor. Although the difference between the value of ${S}_{{I}_{ds}}$ for Temp = 300 K and Temp = 350 K is significant for lower values of *V*_*GS*_, the ${S}_{{I}_{ds}}$ remains almost the same for gate voltages higher than 0.8 V.

As we have emphasized above, the significant benefits of our biosensor lie in its scalability and easier fabrication process. However, since the main electric field of the gate affecting the channel is dominant compared to the fringing field, we expected that conventional DMFETs show better sensitivity than that of our proposed biosensor (at least in simulations). Table 1 presents a comparative evaluation of the performance of various dielectric-modulated biosensors, which have been recently reported. In order to prepare this table, we selected the Gelatin biomolecule with a dielectric constant of 12 as the target for our analysis. We focused on comparing the off-state current sensitivity of the biosensors given by ${S}_{{I}_{off}}\left(\%\right)=\left(\frac{{I}_{off}^{bio}-{I}_{off}^{air}}{{I}_{off}^{air}}\right)\times 100$, which is a critical parameter for their performance evaluation^41^.

**Table 1:** Performance evaluation of some dielectric-modulated biosensor for Gelatin biomolecule.

Ref.	Year	Architecture	Material	${S}_{{I}_{off}}$	*V*_*Bias*_ (V)
^38^	2019	Double gate junction-less TFET	Si	~ 100	1.2
^39^	2021	Extended gate HTFET	InGaAs/Si	90	1.5
^40^	2021	FinFET	GaAs_1−x_Sb_x_	98.4	1
^41^	2021	Negative capacitance FinFET	Si	99.99	1
^27^	2023	Vertical dual doping-less tunneling junction	Si	98.58	1.5
This work	2023	Double gate TFET	Si	74.47	1

## Conclusion

We have developed a new type of biosensor that uses the concept of fringe field capacitance. Unlike traditional dielectric-modulated bioTFET, our biosensor has carved cavities in the spacer regions, making gate leakage current no longer a problem. We also depicted that our biosensor is easier to realize and less-challenging to fabricate. We used the Silvaco ATLAS devices simulator to carry out all the numerical simulations. Our evaluation involves assessing the impact of both neutral and charged biomolecules on critical parameters such as drain current sensitivity (${S}_{{I}_{ds}}$) and subthreshold swing sensitivity (*S*_*SS*_). Furthermore, we have investigated the impact of less-analyzed non-idealities, such as traps-assisted tunneling and temperature, on the performance of our bioTFET. Our device’s parameters, including ${S}_{{I}_{ds}}$ = 1.21 × 10^3^, *S*_*SS*_  =  0.365, and ${S}_{{f}_{T}}$ = 1.63 × 10^3^, demonstrate that it can compete with other TFET-based dielectric-modulated biosensors.

## Data Availability

The datasets used and/or analyzed during the current study available from the corresponding author on reasonable request.
